# DAILY STRESSORS AND DAILY CONTROL BELIEFS ARE ASSOCIATED WITH DAILY ACCELERATED AGING

**DOI:** 10.1093/geroni/igae098.2103

**Published:** 2024-12-31

**Authors:** Shevaun Neupert, Kathryn Swaim

**Affiliations:** North Carolina State University, Raleigh, North Carolina, United States; North Carolina State University, Raleigh, North Carolina, United States

## Abstract

Exposure to daily stressors is consistently associated with reduced health and well-being, including feeling subjectively older. Experiencing increases in daily perceptions of control, however, are typically associated with increased health and well-being and feeling subjectively younger. The current study focuses on subjective accelerated aging from a daily perspective, capturing day-to-day fluctuations in the perceived rate at which people feel that they are aging. The goal of the current study was to bring together daily stressors and daily perceptions of control to test their independent contributions to the understanding of daily accelerated aging. A sample of 440 U.S. adults aged 50-85 participated in a 14-day daily diary study, reporting on their perceived rate of aging, exposure to daily stressors, and perceptions of daily control beliefs each day. All variables demonstrated significant within-person variability, with ICCs ranging from.60 (rate of aging) to.78 (control beliefs). Daily stressors and daily control beliefs were each uniquely predictive of daily accelerated aging, where daily stressors were positively associated and daily control beliefs were negatively associated with daily accelerated aging. These findings underscore the dynamic and contextual nature of subjective aging metrics and document the within-person coupling between events and beliefs.

